# Supplementary material for: A third type of PETase from the marine Halopseudomonas lineage
Source: Protein Sci. 2025 Sep 17;34(10):e70305. doi: 10.1002/pro.70305 (PMC12442448; doi:10.1002/pro.70305)
Supplement: Supplementary file 1 — FIGURE S1. Functional screening of target proteins via plate‐based assay. FIGURE S2. SDS‐PAGE gels of HaloPETase purifications. FIGURE S3. Residual activity of HaloPETase1 tested via pNPB hydrolysis depending on NaCl concentration, time and temperature. FIGURE S4. Effect of single mutations in the subsite I of HaloPETase1. FIGURE S5. Sequence identity matrix of pairwise alignments with candidate PETases from the high bit‐score group. FIGURE S6. Sequence identity matrix of pairwise alignments with candidate PETases from the low bit‐score group. FIGURE S7. MSA of low bit‐score PETases including HaloPETase1 to 4 and PmC. FIGURE S8. Phylogenetic tree of PETases from the PAZy database, here characterized, and putative PETases from the Halopseudomonas lineage. FIGURE S9. Salt‐ and temperature‐dependent PET‐degradation by HaloPETase2 to 6 from low and high bit‐score groups. FIGURE S10. Logo plot of important regions in HaloPETases from the lower bit‐score group. FIGURE S11. PET‐film characterization by DSC. TABLE S1. Functional plate‐based screening of recombinant target proteins. TABLE S2. BLASTp hits for HaloPETase1 in the databases PDB and UniProtKB. TABLE S3. Crystallographic data collection and refinement statistics. TABLE S4. Results of HaloPETase1 homolog mining in Halopseudomonas using pHMM analysis. TABLE S5. Primer pairs for the introduction of single mutations in HaloPETase‐1 by site‐directed mutagenesis. [file PRO-34-e70305-s001.pdf]

Supplementary Information

A third type of PETase from the marine *Halopseudomonas* lineage

Onur Turak[1,2], Andreas Gagsteiger[1], Ashank Upadhyay[3], Mark Kriegel[1], Peter Salein[1], Stefanie Böhnke-Brandt[2], Seema Agarwal[3], Erik Borchert[2*] & Birte Höcker[1*]

[1] Department of Biochemistry, University of Bayreuth, Bayreuth, Germany

[2] RD3 Marine Symbioses, GEOMAR Helmholtz Centre for Ocean Research Kiel, Kiel, Germany

[3] Advanced Sustainable Polymers, Macromolecular Chemistry II, University of Bayreuth, Bayreuth, Germany

* Corresponding authors: birte.hoecker@uni-bayreuth.de; eborchert@geomar.de

List of Supplementary Figures:

- **Figure S1**: Functional screening of target proteins via plate-based assay
- **Figure S2**: SDS-PAGE gels of *Halo*PETase purifications
- **Figure S3**: Residual activity of *Halo*PETase1 tested via *p*NPB hydrolysis depending on NaCl concentration, time and temperature
- **Figure S4:** Effect of single mutations in the subsite I of *Halo*PETase1
- **Figure S5:** Sequence identity matrix of pairwise alignments with candidate PETases from the high bit-score group
- **Figure S6:** Sequence identity matrix of pairwise alignments with candidate PETases from the low bit-score group
- **Figure S7:** MSA of low bit-score PETases including *Halo*PETase1 to 4 and *Pm*C
- **Figure S8:** Phylogenetic tree of PETases from the PAZy database, here characterized, and putative PETases from the *Halopseudomonas* lineage
- **Figure S9:** Salt- and temperature-dependent PET-degradation by *Halo*PETase2 to 6 from low and high bit-score groups
- **Figure S10:** Logo plot of important regions in *Halo*PETases from the lower bit-score group
- **Figure S11:** PET-film characterization by DSC

List of Supplementary Tables:

- **Table S1**: Functional plate-based screening of recombinant target proteins
- **Table S2:** BLASTp hits for *Halo*PETase1 in the databases PDB and UniProtKB
- **Table S3:** Crystallographic data collection and refinement statistics
- **Table S4:** Results of *Halo*PETase1 homolog mining in *Halopseudomonas* using pHMM analysis
- **Table S5:** Primer pairs for the introduction of single mutations in *Halo*PETase-1 by site-directed mutagenesis

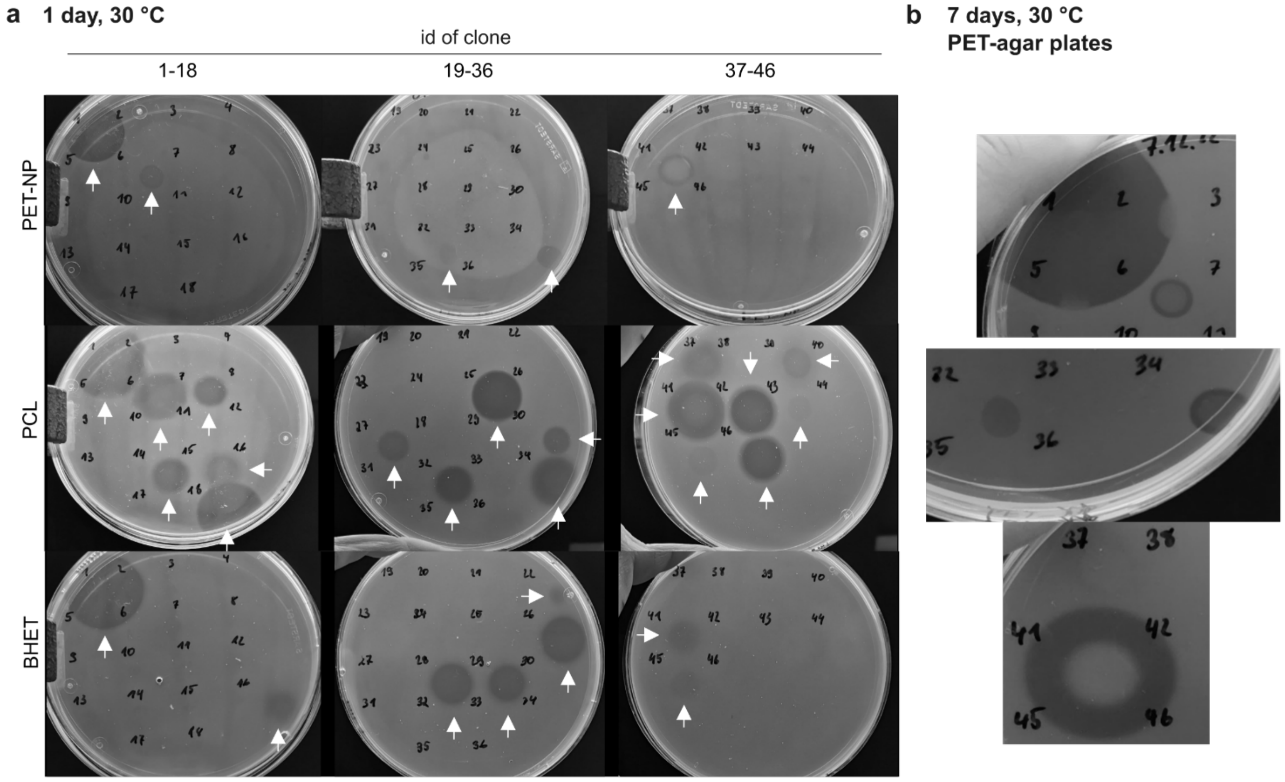

Figure S1: Functional screening of target proteins via plate-based assay. PBS-agar (1.5 % [w/v] agar) plates were supplemented with either PET-NP, PCL or BHET and purified enzyme solutions were added on the agar surface. **a)** Zone of clearances on all plates were observed after 1 day **b)** and on PET-NP after 7 days of incubation at 30 °C. IDs next to the spots and above in **a** and **b** represent an individual recombinant target protein as shown in Table S1.

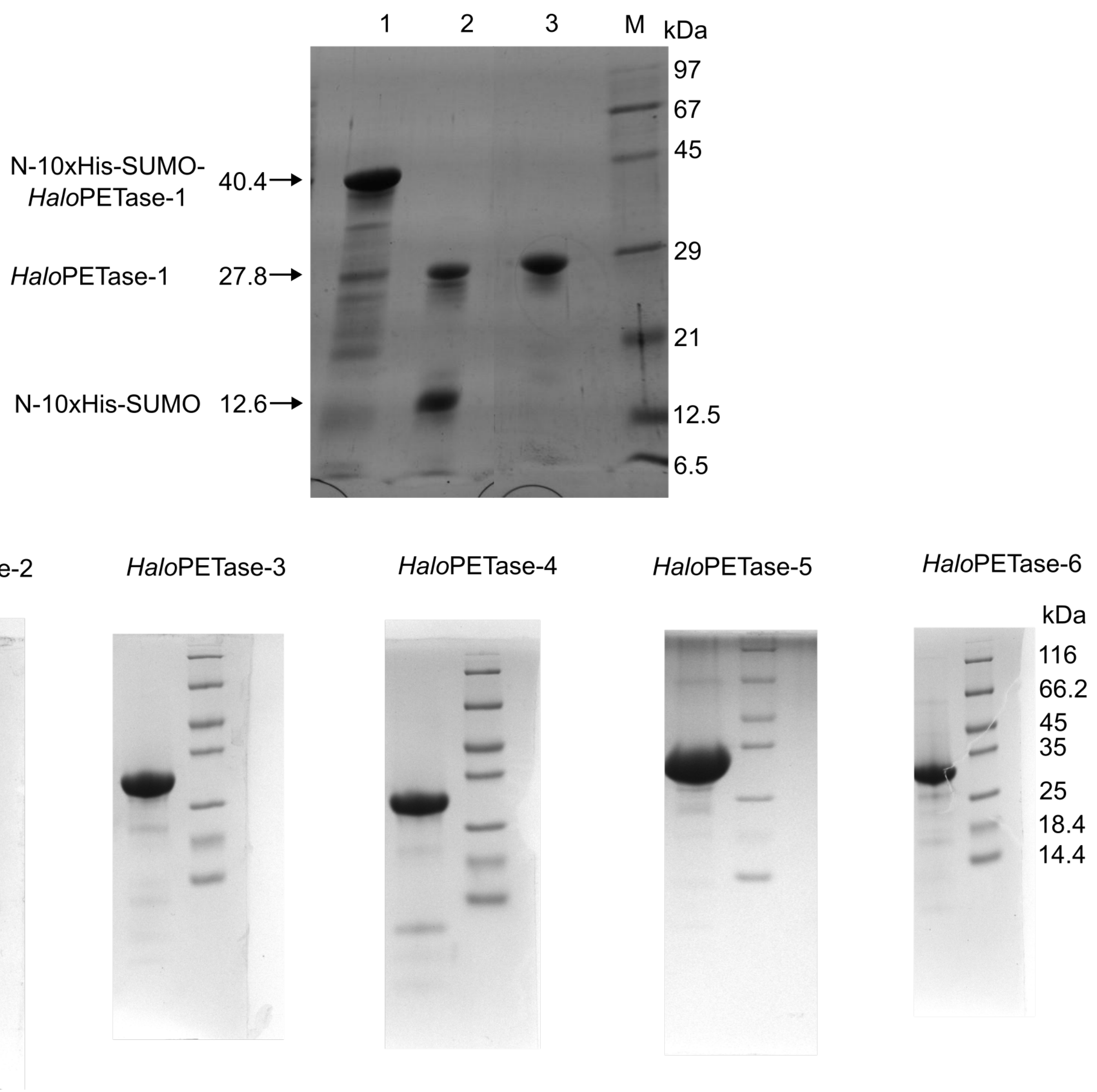

Figure S2: SDS-PAGE gels of *Halo*PETase purifications. Upper gel: Purification of N-10xHis-SUMO-tagged *Halo*PETase-1 by Ni-IMAC (lane 1), SenP2-digestion and dialysis for the removal of N-10xHis-SUMO-tag (lane 2), reverse Ni-IMAC and SEC (lane 3) to obtain truncated and purified *Halo*PETase1. Lower gels: Final purified protein solutions of *Halo*PETases2 to 6.

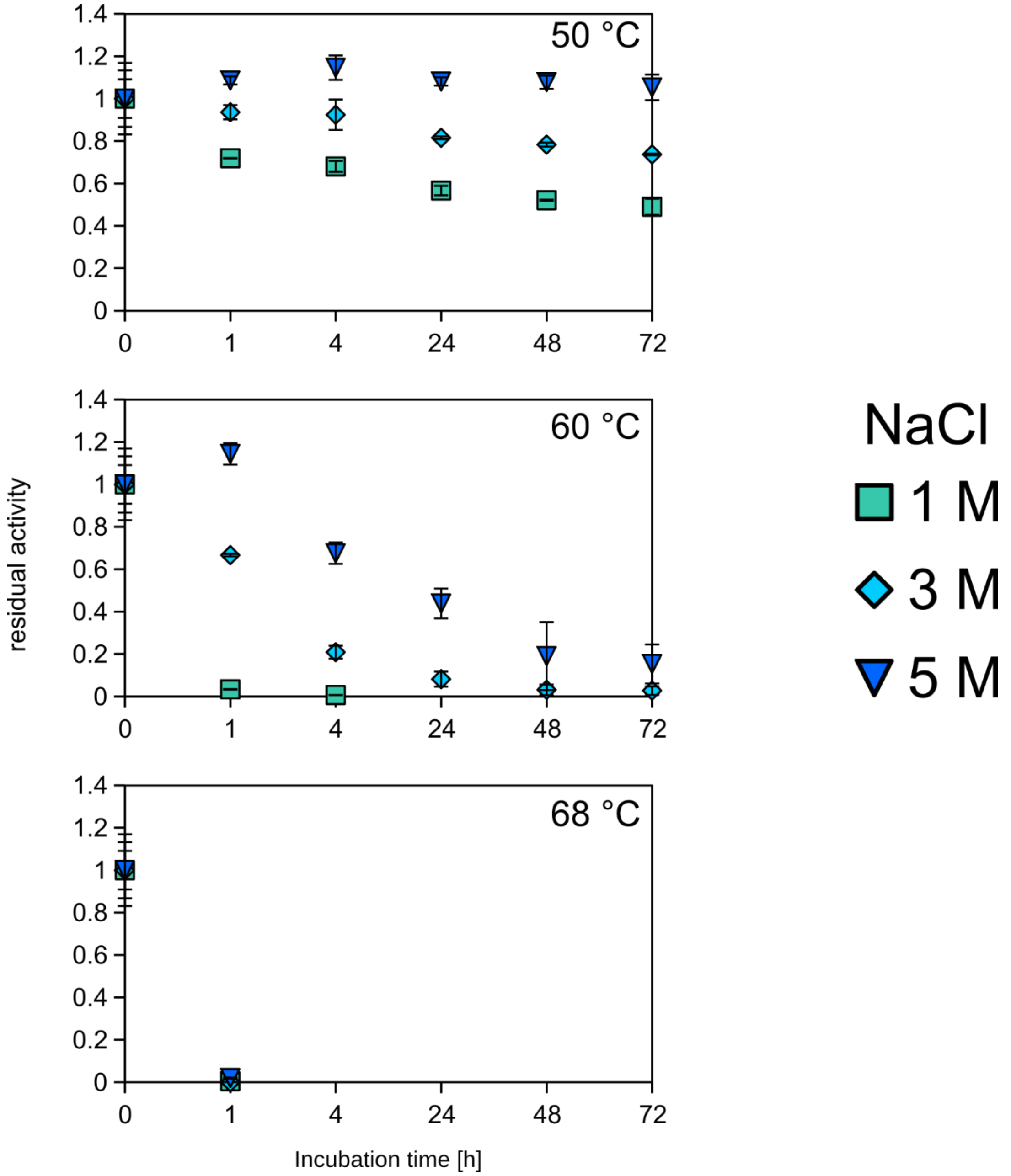

Figure S3: Residual activity of *Halo*PETase1 tested via *p*NPB hydrolysis depending on NaCl concentration, time and temperature. The residual activity was assessed by measuring the absorbance of the hydrolysis product *p*NP at 405 nm after incubation under specific salt and temperature conditions and normalizing the change at A_{405nm} over time to the initial rate at 0 hours. Enzyme solutions were prepared with 5 µM concentration in the respective condition and incubated for up to 72 h. Subsequently, incubated solutions were diluted 100-fold in reaction solution, containing 20 mM sodium phosphate, 150 mM NaCl with pH 7.4 and 1 mM *p*NPB.

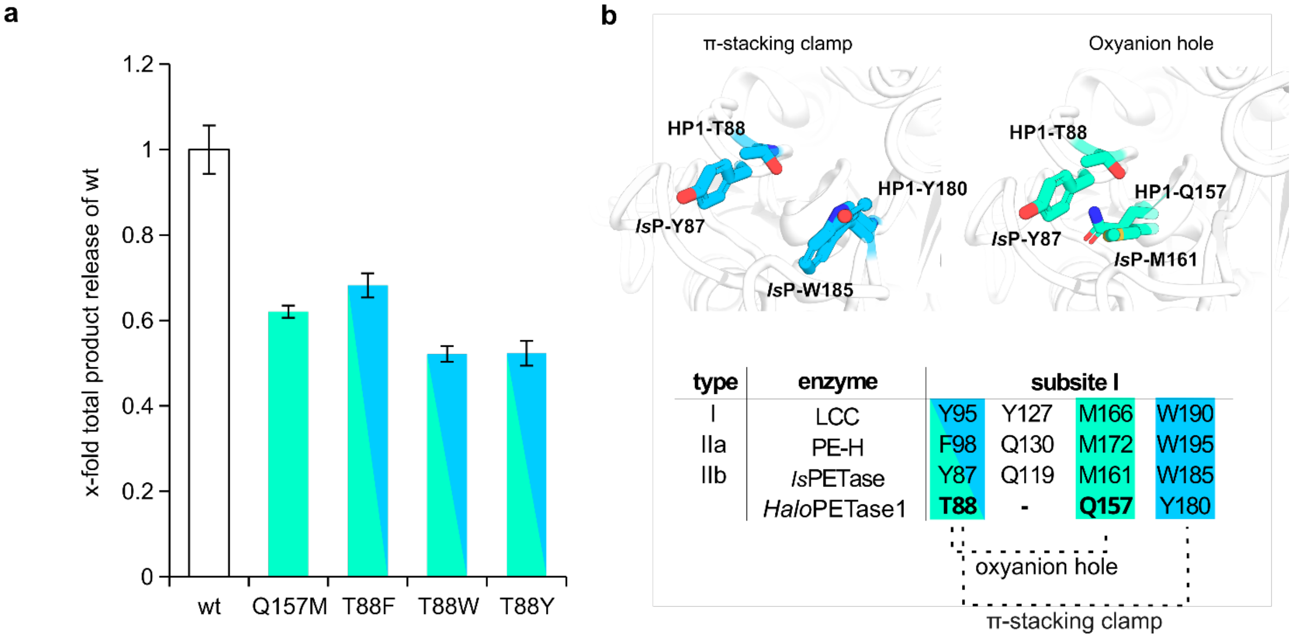

type	enzyme	subsite I			
I	LCC	Y95	Y127	M166	W190
IIa	PE-H	F98	Q130	M172	W195
IIb	*Is*PETase	Y87	Q119	M161	W185
	*Halo*PETase1	**T88**	-	**Q157**	Y180

Figure S4: Effect of single mutations in the subsite I of *Halo*PETase1. **a**) PET-degradation by *Halo*PETase1 (wt) and its variants in a PET-coated well-plate assay. T88 was substituted with an aromatic amino acid (T88F, T88W, T88Y) to restore the canonical π-stacking clamp. Q157 was substituted by methionine (Q157M) to obtain the representative occupation in the oxyanion hole, as in other PETases. Reaction buffer composition: 20 mM NaP_i, 3 M NaCl, pH 7.4. The reactions were incubated at 50 °C for 20 h. **b**) Aligned subsite I of *Halo*PETase1 (HP1) and *Is*PETase. Amino acids corresponding to the oxyanion hole (mint) or π-stacking clamp (blue) are shown as sticks. Subsite I amino acid occupation from type I, IIa, IIb and *Halo*PETase1 is shown in the table, where significant substitutions in *Halo*PETase1 are highlighted with bold letters.

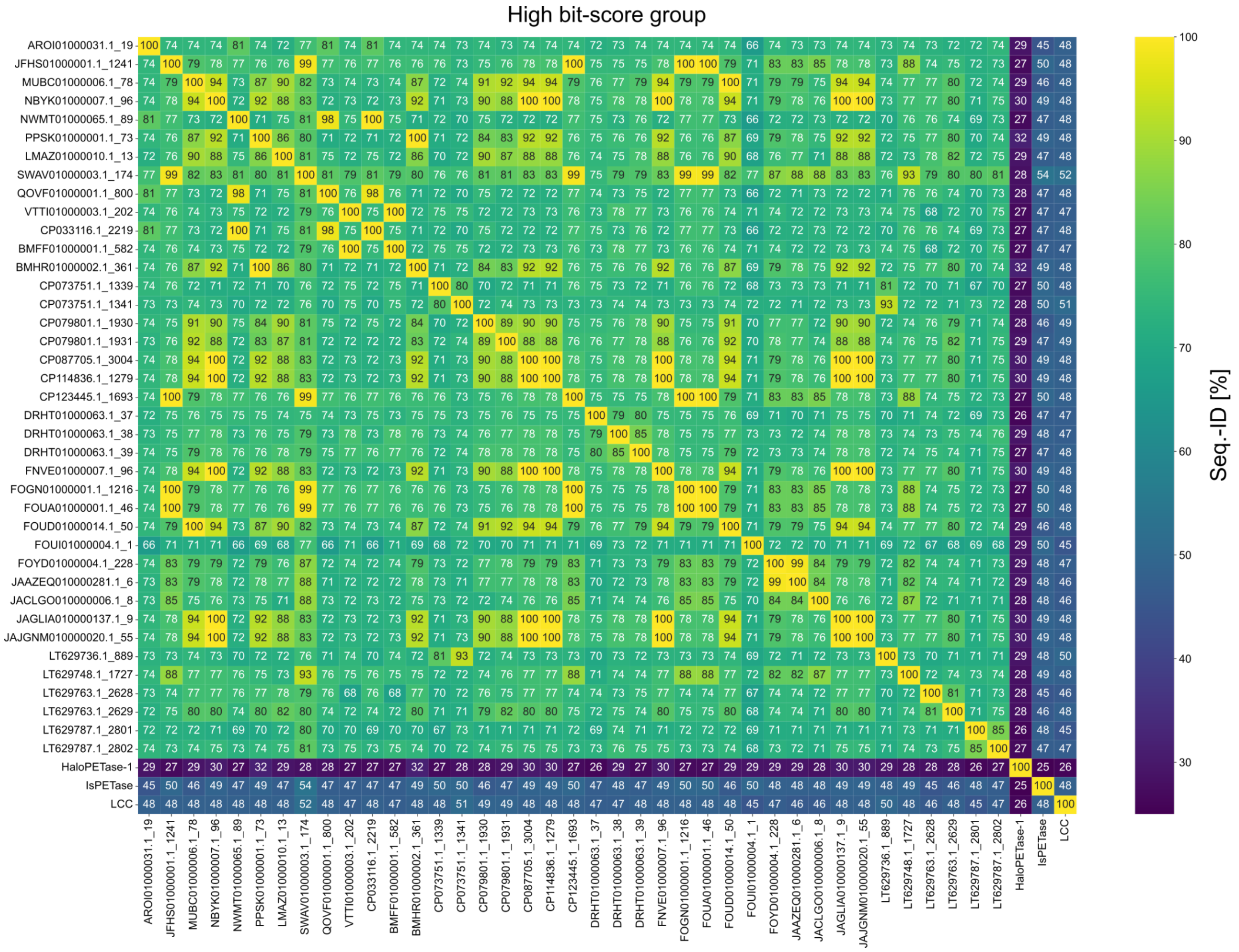

Figure S5: Sequence identity matrix of pairwise alignments with candidate PETases from the high bit-score group. All aligned sequences were included in the phylogenetic tree in Figure 3a. Pairwise alignments were performed by ProbCons and sequence identities were inferred from ClustalX[5].

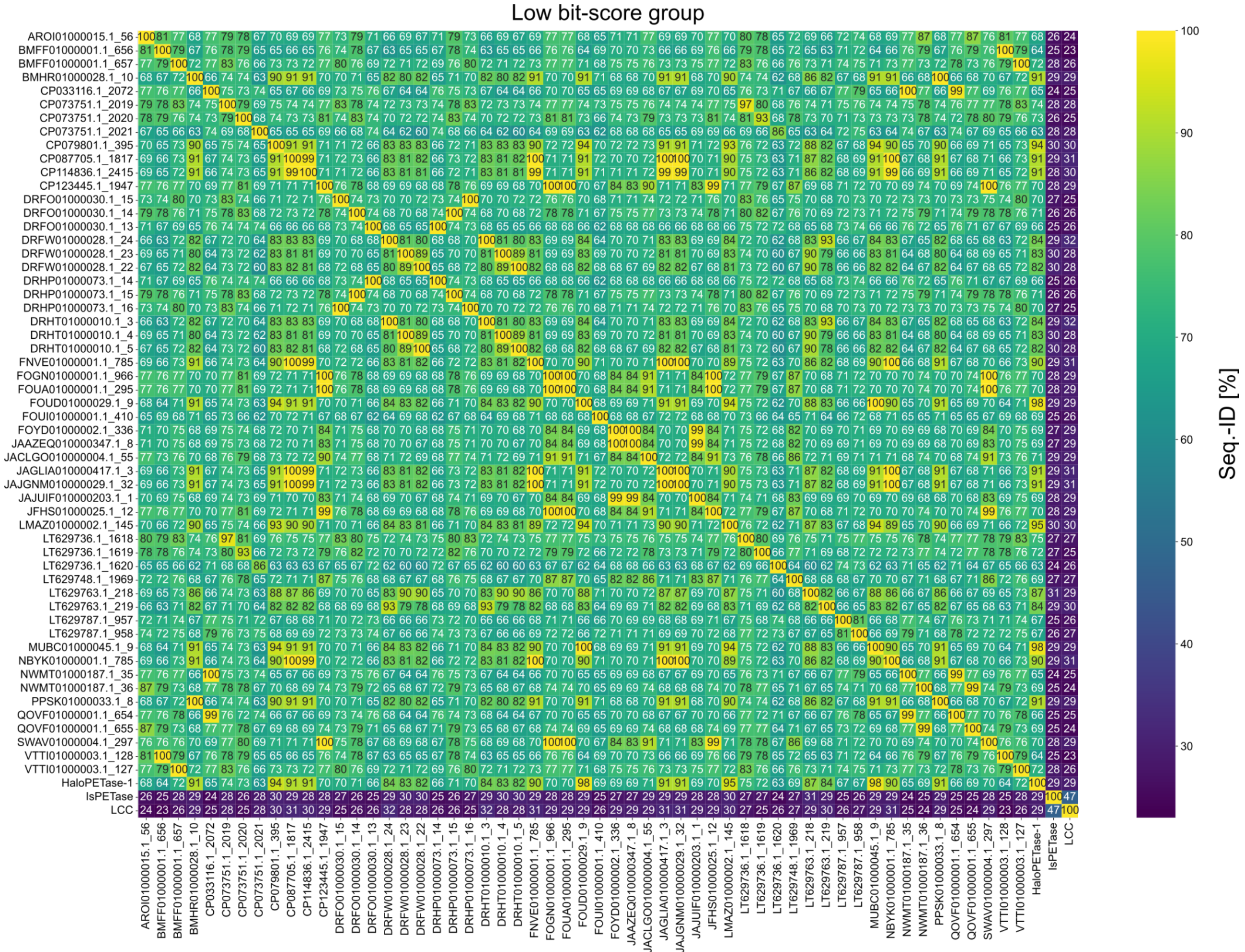

Figure S6: Sequence identity matrix of pairwise alignments with candidate PETases from the low bit-score group. All aligned sequences were included in the phylogenetic tree in Figure 3a. Pairwise alignments were performed by ProbCons and sequence identities were inferred from ClustalX[5].

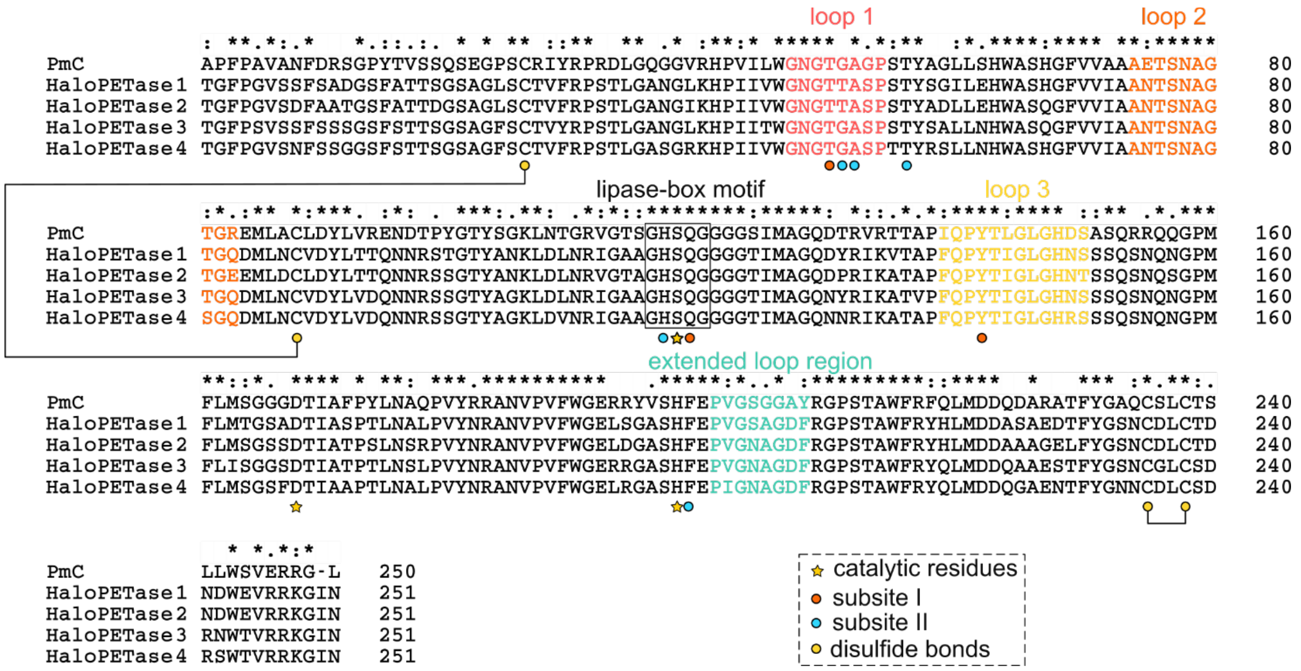

Figure S7: MSA of low bit-score PETases including *Halo*PETase1 to 4 and *Pm*C. Numbers at the end of each line represent the sequence position of the last amino acid of the corresponding protein from each line. The extent of conservation at each position is indicated by '*' for identical, ':' for highly similar or '.' less similar residues, according to the Gonnet PAM 250 scoring matrix[6]. Colored circle or star symbols indicate potentially important and catalytic residues at the active site, respectively. Connected yellow circles represent cysteines forming a disulfide bond. Disulfide bonds from *Halo*PETase2 to 4 are inferred from *Halo*PETase1. The extended loop region (turquoise-colored letters) was inferred from Joo *et al*.[7]. Loops characterized in this work (loop 1 to 3) are colored according to Figure 3.

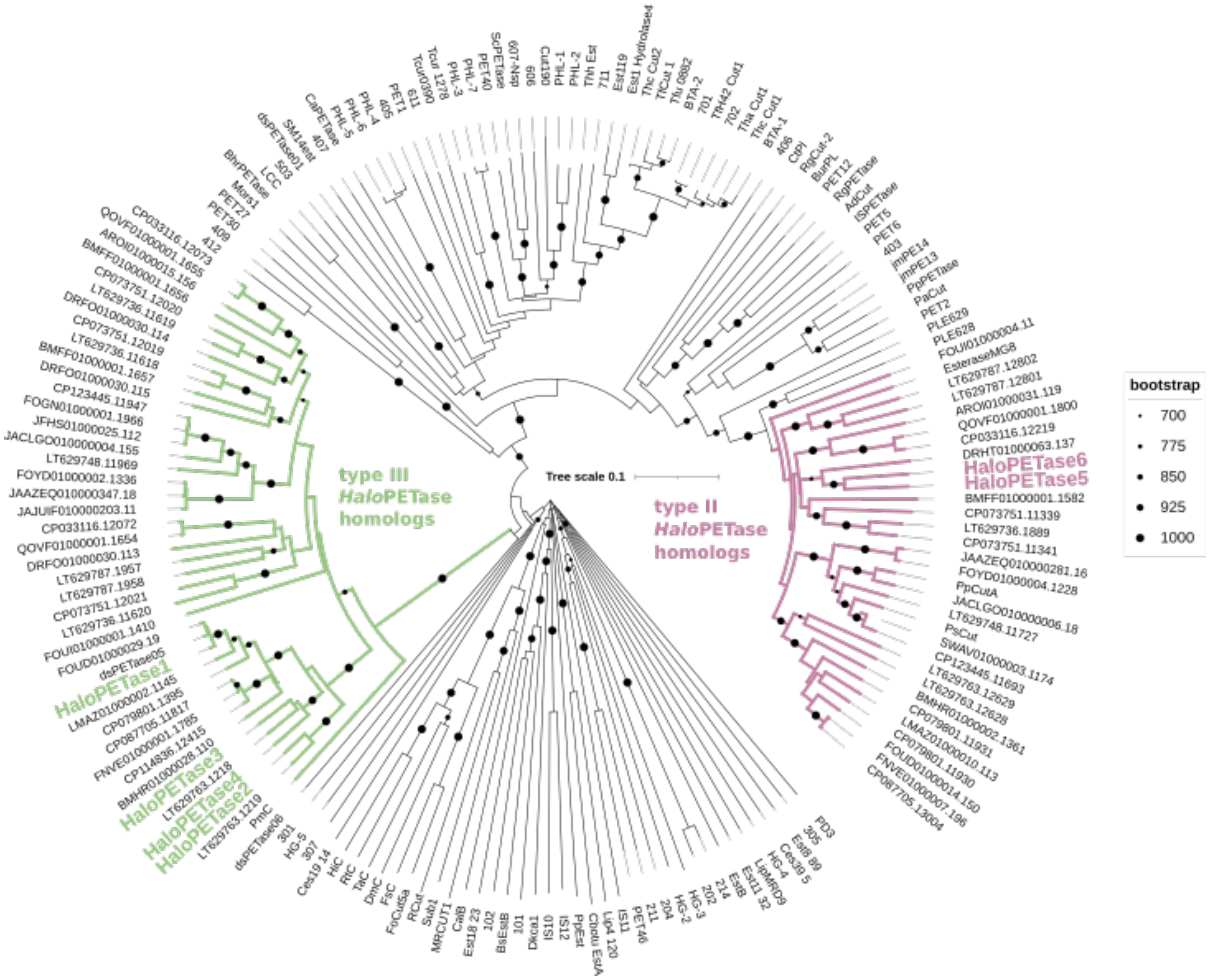

Figure S8: Type III *Halo*PETases form a separate branch in phylogenetic analysis of known and putative PETases. The tree was constructed with PETases from the PAZy database, plus here characterized and putative PETases from the *Halopseudomonas* lineage (see also table S4). *Halo*PETases1 to 6 are highlighted in bold. Initially, a full-length protein sequence alignment using ClustalX was performed and a neighborhood-joining tree with 1000 bootstraps was constructed subsequently. The tree was illustrated using the interactive tree of life tool (https://itol.embl.de/).

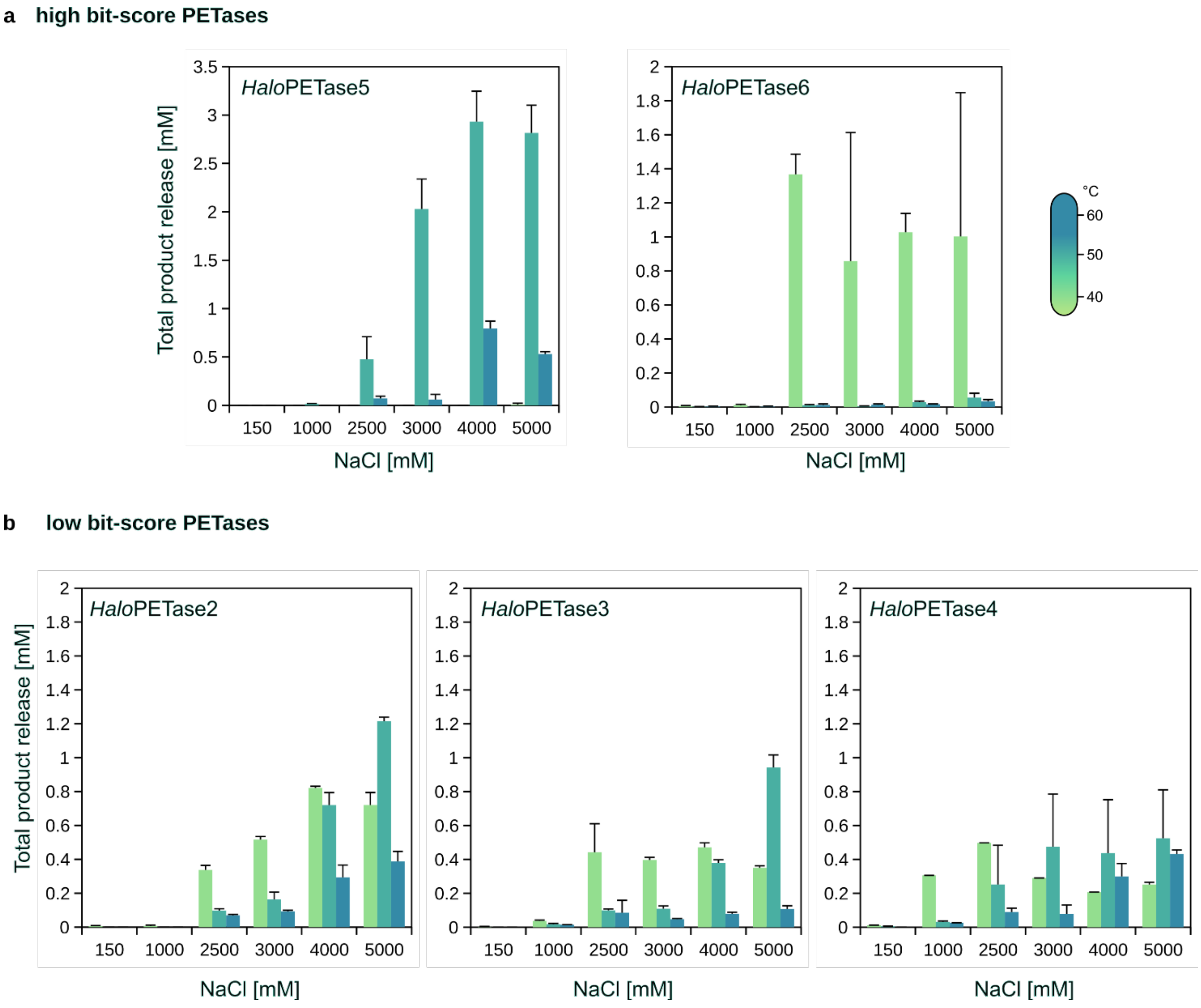

Figure S9: Salt- and temperature-dependent PET-degradation by *Halo*PETase2 to 6 from low and high bit-score groups. Degradation experiments were performed with the PET-coated assay according to Weigert *et al.* (see Methods section). The buffer for each experiment was 20 mM NaP_i, pH 7.4 with variable NaCl-concentrations. Protein concentrations were selected according to preliminary testing for optimal concentration as follows: **a)** high bit-score *Halo*PETases: *Halo*PETase5 550 nM; *Halo*PETase6: 50 nM. **b)** low bit-score *Halo*PETases: *Halo*PETase2 500 nM; *Halo*PETase3 550 nM; *Halo*PETase4 150 nM. Error-bars indicate standard deviations of triplicate measurements.

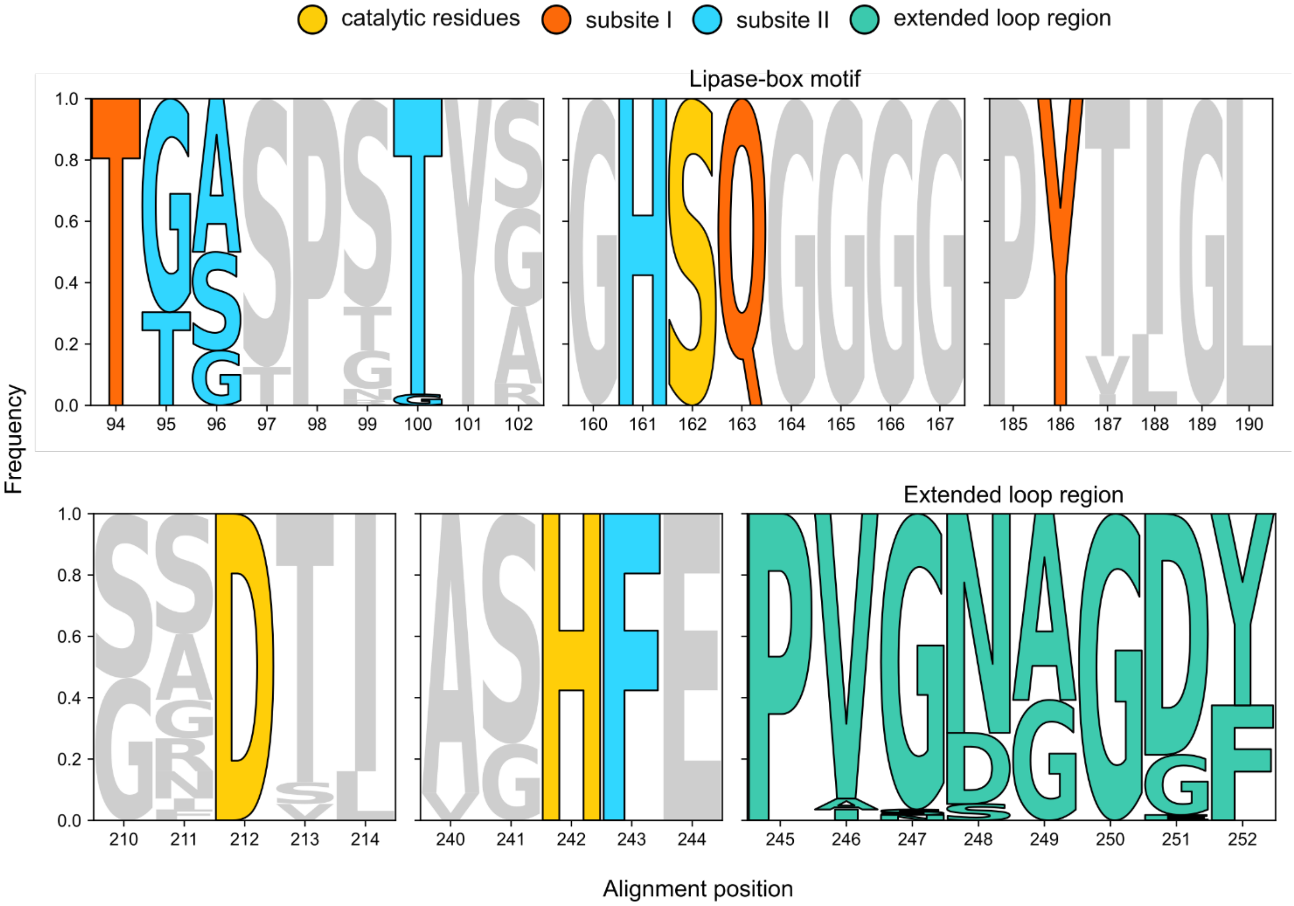

Figure S10: Logo plot of important regions in *Halo*PETases from the lower bit-score group. Important residues and regions for PETase activity according to Joo *et al.*[7]. The logo-plot was generated using full-length sequences of putative low bit-score PETase sequences from *Halopseudomonas*, as listed in Table S4.

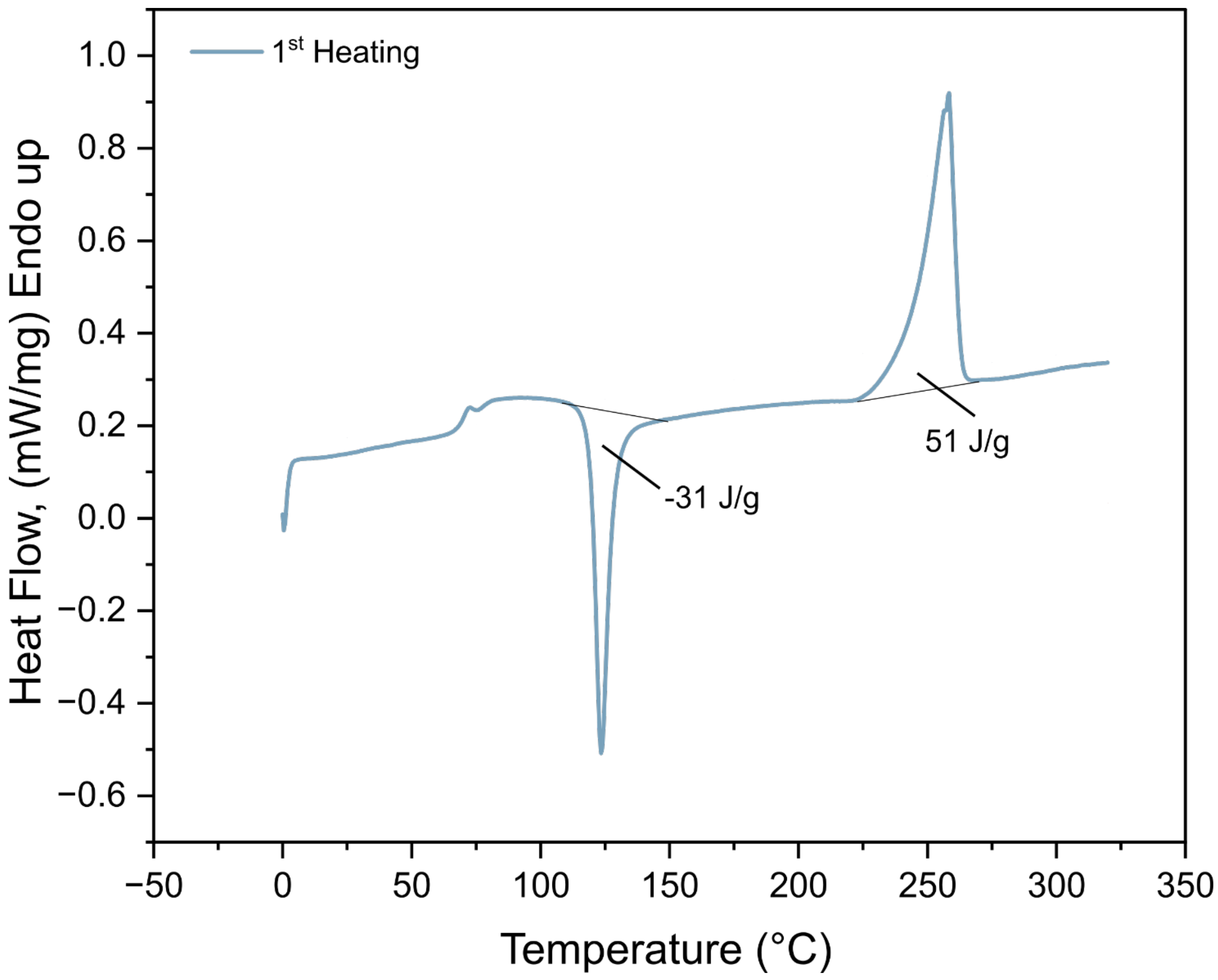

Figure S11: PET-film characterization by DSC. Calculation of crystallinity was performed using the first heating curve. More specifically, the heat of crystallization (31 J/g) was subtracted from the heat of fusion (45 J/g) and divided by the heat of fusion for 100 % crystalline PET (see Methods).

Table S1: Functional plate-based screening of recombinant target proteins. The purified protein IDs correspond to the tested proteins in Figure S1. Target protein ID 1 corresponds to the positive control, IDs 2-5 to LCC(ICCG) and negative controls, i.e. GSGSGS peptide sequence attached to one of the possible purification and/or solubility tags. A '+' represents qualitatively detected zone of clearance and '-' no detection of the same.

Purified protein ID	Target protein	Tag	PCL	BHET	PET-NP
1	LCC(ICCG)	C-6xHis	+	+	+
2	GSGSGS	N-10xHis	-	-	-
3	GSGSGS	C-6xHis	-	-	-
4	GSGSGS	N-10xHis-MBP	-	-	-
5	GSGSGS	N-10xHis-SUMO	-	-	-
6	***Halo*PETase1**	N-10xHis	+	-	+
7	ANW_6_2	N-10xHis	+	-	-
8	PSW_64_1	N-10xHis	-	-	-
9	ION_47_1	N-10xHis	-	-	-
10	ASW_18_1	N-10xHis	+	-	-
11	IOS_14_1	N-10xHis	+	-	-
12	PSE_95_1	N-10xHis	-	-	-
13	P01_A02_3_1	N-10xHis	-	-	-
14	PSE_112_1	N-10xHis	+	-	-
15	ANW_30_1	N-10xHis	+	-	-
16	PSE_138_1	C-6xHis	+	+	-
17	***Halo*PETase1**	C-6xHis	-	-	-
18	ANW_6_2	C-6xHis	+	-	-
19	IOS_57_1	C-6xHis	-	-	-
20	ION_47_1	C-6xHis	-	-	-
21	ASW_18_1	C-6xHis	-	-	-
22	IOS_14_1	C-6xHis	-	+	-
23	PSE_95_1	C-6xHis	-	-	-
24	P01_A02_3_1	C-6xHis	-	-	-
25	PSE_112_1	C-6xHis	+	-	-
26	SWF_bin8_13	C-6xHis	-	+	-
27	ANW_30_1	C-6xHis	+	-	-
28	IOS_26_1	C-6xHis	-	+	-
29	PON_52_1	C-6xHis	-	+	-
30	PSE_138_1	N-10xHis-MBP	-	+	-
31	PSE_120_1	N-10xHis	-	-	-
32	PSW_62_1	N-10xHis	+	-	+
33	PSE_130_1	N-10xHis	+	-	-
34	ASW_29_1	N-10xHis	+	-	+
35	ANW_6_1	N-10xHis	-	-	-
36	PSE_120_1	C-6xHis	+	-	-
37	PSW_62_1	C-6xHis	+	-	+
38	PSE_130_1	C-6xHis	-	-	-
39	ASW_29_1	C-6xHis	+	-	-
40	ANW_6_1	C-6xHis	-	-	-
41	***Halo*PETase1**	N-10xHis-SUMO	+	+	+
42	ANW_6_2	N-10xHis-SUMO	+	-	-
43	ASW_18_1	N-10xHis-SUMO	+	-	-
44	PSE_95_1	N-10xHis-SUMO	-	-	-
45	P01_A02_3_1	N-10xHis-SUMO	+	+	-
46	PSE_112_1	N-10xHis-SUMO	+	-	-

Table S2: BLASTp hits for *Halo*PETase1 in the databases PDB and UniProtKB. The analysis was performed on August 6th, 2024. The hit with highest sequence identity, *Pseudomonas mendocina* cutinase, is highlighted in bold.

Entry	Description	Identity (%)	Similarity (%)	Seq. coverage	E-value	Database
**2FX5**	***Pseudomonas mendocina* cutinase**	**62.4**	**75.3**	**255**	**4.22E-111**	**PDB**
6AID	*Thermobifida alba* cutinase	33.5	48.9	188	1.7E-17	PDB
3VIS	*Thermobifida alba* AHK119 Est119	33.5	48.9	188	2.22E-17	PDB
8IAN	*Caldimonas taiwanensis* *Ct*PL-H210S/F214I	32.9	50.0	140	3.22E-12	PDB
F7IX06.1	*Thermobifida alba* cut2	33.5	48.9	188	8.35E-17	UniProtKB
G9BY57.1	Leaf-branch compost cutinase	28.6	42.9	266	6.23E-16	UniProtKB
E9LVH8.1	*Thermobifida cellulosilytica* Cutinase 1	30.2	48.4	182	1.37E-13	UniProtKB
D4Q9N1.2	*Thermobifida alba* Cutinase est1	29.6	47.1	223	1.89E-13	UniProtKB

Table S3: Crystallographic data collection and refinement statistics.

**Data collection**	
Beamline	PETRA III P13
Wavelength [Å]	0.9763
Space group	$P\ 2_1 2_1 2_1$
Unit cell [Å, °]	a = 52.3 b = 70.5 c = 72.5<br>α = β = γ = 90
Resolution range [Å]	35.26 - 1.16<br>(1.19 - 1.16)*
Unique reflections	93276 (6131)
Multiplicity	6.6 (6.6)
Completeness [%]	99.9 (99.8)
R-meas [%]	6.6 (44.5)
<I/σI>	15.86 (4.17)
$CC_{1/2}$	0.999 (0.929)
*CC**	1 (0.981)
Wilson *B*-factor [Å^2]	8.8
**Refinement**	
R_{work}/R_{free} [%]	11.0/12.9
No. of atoms (non-H)<br>macromolecules<br>ligands<br>solvent	 2025<br>196<br>185
RMSD from ideal geometry<br>bonds [Å]<br>angles [°]	 0.005<br>0.98
Ramachandran statistics<br>favored [%]<br>outliers [%]	 97.7<br>0.00
Clashscore	3.9
Average *B* [Å^2]<br>macromolecules<br>ligands<br>solvent	13.4**<br>10.3<br>29.4<br>30.6

* Statistics for the highest-resolution shell are shown in parentheses.

** Refinement of individual anisotropic B-factors for all atoms excluding hydrogens

Table S4: Results of *Halo*PETase1 homolog mining in *Halopseudomonas* using pHMM analysis. The pHMM analysis was performed in *Halopseudomonas* genome assemblies, which can be found by their NCBI Genome GenBank[1] accession IDs. Corresponding hits are shown in the 'Protein designation' column, whereby the IDs correspond to the position of the protein on the specific contig. Hit sequences were affiliated with the low bit-score group if the respective bit-score was lower than 200. Otherwise, hits belong to the high bit-score group. Length in number of amino acids in one hit sequence corresponds to a full sequence including possible N-terminal signal peptide sequence. Biochemically characterized hits are referred to a publication or to this work in the column 'Characterized'. Characterized proteins were verified through a BLASTp[2] search against non-redundant protein databases on September 11, 2024.

GenBank genome accession	Protein designation	Affiliation	Length (aa)	Bit-score	Bit-score group	PETase type	Characterized
GCA_020781875.1	JAJGNM010000029.1 32	H. aestusnigri FXH-240	290	143.8	Low	Putative III	-
GCA_020781875.1	JAJGNM010000020.1 55	H. aestusnigri FXH-240	304	395.2	High	Putative IIa	-
GCA_021184005.1	CP087705.1 1817	H. aestusnigri GOM5	290	143.8	Low	Putative III	-
GCA_021184005.1	CP087705.1 3004	H. aestusnigri GOM5	304	395.2	High	Putative IIa	-
GCA_023143355.1	JAGLIA010000417.1 3	H. aestusnigri RS 2 23	290	143.8	Low	Putative III	-
GCA_023143355.1	JAGLIA010000137.1 9	H. aestusnigri RS 2 23	304	395.2	High	Putative IIa	-
GCA_002197985.1	NBYK01000001.1 785	H. aestusnigri VGXO14	289	143.7	Low	Putative III	-
GCA_002197985.1	NBYK01000007.1 96	H. aestusnigri VGXO14	304	396.3	High	IIa	*Haes*_PE-H[3]
GCA_005096325.1	SWAV01000004.1 297	H. bauzanensis SBBB	287	140	Low	Putative III	-
GCA_005096325.1	SWAV01000003.1 174	H. bauzanensis SBBB	196	288.2	High	IIa	*Pbauz*Cut[4]
GCA_000632535.1	JFHS01000025.1 12	H. bauzanensis W13Z2	287	142.8	Low	Putative III	-
GCA_000632535.1	JFHS01000001.1 1241	H. bauzanensis W13Z2	302	398.9	High	IIa	*Pbauz*Cut[4]
GCA_012512195.1	JAAZEQ010000347.1 8	H. formosensis AS09scLD 420	287	143.8	Low	Putative III	-
GCA_012512195.1	JAAZEQ010000281.1 6	H. formosensis AS09scLD 420	304	392.3	High	Putative IIa	-
GCA_029263535.1	JAJUIF010000203.1 1	H. formosensis bin83	287	144.6	Low	Putative III	-
GCA_029263535.1	JAJUIF010000365.1 5	H. formosensis bin83	129	162.2	Low	Putative III	-
GCA_003444685.1	LMAZ01000002.1 145	H. gallaeciensis V113	289	139.3	Low	Putative III	-
GCA_003444685.1	LMAZ01000010.1 13	H. gallaeciensis V113	305	383.3	High	Putative IIa	-
GCA_008365385.1	QOVF01000001.1 655	H. laoshanensis Y22	288	139.8	Low	Putative III	-
GCA_008365385.1	QOVF01000001.1 654	H. laoshanensis Y22	287	146.3	Low	Putative III	-
GCA_008365385.1	QOVF01000001.1 800	H. laoshanensis Y22	310	402.2	High	Putative IIa	-
GCA_002903165.1	PPSK01000033.1 8	H. oceani DSM 100277	288	132.8	Low	Putative III	-
GCA_002903165.1	PPSK01000001.1 73	H. oceani DSM 100277	303	395.2	High	Putative IIa	-

GCA_001989375.1	MUBC01000045.1 9	H. pachastrellae CCUG 46540	289	139.6	Low	Putative III	-
GCA_001989375.1	MUBC01000006.1 78	H. pachastrellae CCUG 46540	304	387	High	Putative IIa	-
GCA_015661625.1	DQVS01000080.1 1	H. pachastrellae SZUA-1527	127	154.5	Low	Putative III	-
GCA_002351465.1	NWMT01000187.1 36	H. pelagia 58 153	288	139.2	Low	Putative III	-
GCA_002351465.1	NWMT01000187.1 35	H. pelagia 58 153	286	147.4	Low	Putative III	-
GCA_002351465.1	NWMT01000065.1 89	H. pelagia 58 153	310	404.3	High	Putative IIa	-
GCA_000410875.1	AROI01000015.1 56	H. pelagia CL-AP6	285	139.6	Low	Putative III	-
GCA_000410875.1	AROI01000031.1 19	H. pelagia CL-AP6	307	412.1	High	Putative IIa	-
GCA_009497895.1	CP033116.1 2073	H. pelagia Kongs-67	288	139.2	Low	Putative III	-
GCA_009497895.1	CP033116.1 2072	H. pelagia Kongs-67	286	147.4	Low	Putative III	-
GCA_009497895.1	CP033116.1 2219	H. pelagia Kongs-67	310	404.3	High	Putative IIa	-
GCA_011050335.1	DRFW01000028.1 22	H. sabulinigri HyVt-331	288	139.4	Low	Putative III	-
GCA_011050335.1	DRFW01000028.1 23	H. sabulinigri HyVt-331	290	140.6	Low	Putative III	-
GCA_011050335.1	DRFW01000028.1 24	H. sabulinigri HyVt-331	289	148.2	Low	Putative III	-
**GCA_011053055.1**	**DRHT01000010.1 5**	**H. sabulinigri HyVt-376**	**288**	**139.4**	**Low**	**III**	**This work**
**GCA_011053055.1**	**DRHT01000010.1 4**	**H. sabulinigri HyVt-376**	**290**	**140.6**	**Low**	**III**	**This work**
**GCA_011053055.1**	**DRHT01000010.1 3**	**H. sabulinigri HyVt-376**	**289**	**148.2**	**Low**	**III**	**This work**
GCA_011053055.1	DRHT01000063.1 37	H. sabulinigri HyVt-376	300	385.5	High	Putative IIa	-
**GCA_011053055.1**	**DRHT01000063.1 39**	**H. sabulinigri HyVt-376**	**301**	**388.9**	**High**	**Putative IIa**	**This work**
**GCA_011053055.1**	**DRHT01000063.1 38**	**H. sabulinigri HyVt-376**	**305**	**391.2**	**High**	**Putative IIa**	**This work**
GCA_008641105.1	VTTI01000003.1 127	H. salina XCD-X85	293	134.2	Low	Putative III	-
GCA_008641105.1	VTTI01000003.1 128	H. salina XCD-X85	291	134.6	Low	Putative III	-
GCA_008641105.1	VTTI01000003.1 202	H. salina XCD-X85	308	386.7	High	Putative IIa	-
GCA_027474385.1	CP114836.1 2415	H. sp MFKK-1	289	142.3	Low	Putative III	-
GCA_027474385.1	CP114836.1 1279	H. sp MFKK-1	304	395.2	High	Putative IIa	-
GCA_021545785.1	CP079801.1 395	H. sp RR6	289	147.9	Low	Putative III	-
GCA_021545785.1	CP079801.1 1931	H. sp RR6	305	387.3	High	Putative IIa	-
GCA_021545785.1	CP079801.1 1930	H. sp RR6	304	387.6	High	Putative IIa	-
GCA_029866945.1	CP123445.1 1947	H. sp SMJS2	287	140	Low	Putative III	-
GCA_029866945.1	CP123445.1 1693	H. sp SMJS2	302	398.9	High	Putative IIa	*Pbauz*Cut[4]
GCA_014219065.1	JACLGO010000004.1 55	H. xiamenensis PX1	285	148.2	Low	Putative III	-
GCA_014219065.1	JACLGO010000006.1 8	H. xiamenensis PX1	302	403.8	High	Putative IIa	-

GCA_011050525.1	DRFO01000030.1 14	H. xinjiangensis HyVt-324	287	139.2	Low	Putative III	-
GCA_011050525.1	DRFO01000030.1 15	H. xinjiangensis HyVt-324	289	142.8	Low	Putative III	-
GCA_011053075.1	DRHP01000073.1 14	H. xinjiangensis HyVt-372	286	132.5	Low	Putative III	-
GCA_011053075.1	DRHP01000073.1 15	H. xinjiangensis HyVt-372	287	139.2	Low	Putative III	-
GCA_011053075.1	DRHP01000073.1 16	H. xinjiangensis HyVt-372	289	142.8	Low	Putative III	-
GCA_011050525.1	DRFO01000030.1 13	H. xinjiangensis HyVt-324	286	132.5	Low	Putative III	-
GCA_900108005.1	FNVE01000001.1 785	P. aestusnigri CECT 8317	289	143.7	Low	Putative III	-
GCA_900108005.1	FNVE01000007.1 96	P. aestusnigri CECT 8317	304	396.3	High	IIa	*Haes*_PE-H[3]
GCA_900114735.1	FOUA01000001.1 295	P. bauzanensis CGMCC 1.9095	287	142.8	Low	Putative III	-
GCA_900114735.1	FOUA01000001.1 46	P. bauzanensis CGMCC 1.9095	302	398.9	High	IIa	*Pbauz*Cut[4]
GCA_900111225.1	FOGN01000001.1 966	P. bauzanensis DSM 22558	287	142.8	Low	Putative III	-
GCA_900111225.1	FOGN01000001.1 1216	P. bauzanensis DSM 22558	302	398.9	High	IIa	*Pbauz*Cut[4]
GCA_900115905.1	FOYD01000002.1 336	P. formosensis JCM 18415	287	144.4	Low	Putative III	-
GCA_900115905.1	FOYD01000004.1 228	P. formosensis JCM 18415	304	395.1	High	Putative IIa	-
GCA_900105005.1	LT629748.1 1969	P. litoralis 2SM5	287	145.1	Low	Putative III	-
GCA_900105005.1	LT629748.1 1727	P. litoralis 2SM5	302	401.7	High	Putative IIa	-
GCA_020025155.1	CP073751.1 2021	P. nanhaiensis SCS 2-3	287	126.5	Low	Putative III	-
GCA_020025155.1	CP073751.1 2020	P. nanhaiensis SCS 2-3	286	137.7	Low	Putative III	-
GCA_020025155.1	CP073751.1 2019	P. nanhaiensis SCS 2-3	287	154	Low	Putative III	-
GCA_020025155.1	CP073751.1 1339	P. nanhaiensis SCS 2-3	300	387.2	High	Putative IIa	-
GCA_020025155.1	CP073751.1 1341	P. nanhaiensis SCS 2-3	302	412.4	High	Putative IIa	-
GCA_014641295.1	BMHR01000028.1 10	P. oceani CGMCC 1.15195	288	132.8	Low	Putative III	-
GCA_014641295.1	BMHR01000002.1 361	P. oceani CGMCC 1.15195	303	395.2	High	Putative IIa	-
GCA_900114765.1	FOUD01000029.1 9	P. pachastrellae JCM 12285	289	139.6	Low	Putative III	-
GCA_900114765.1	FOUD01000014.1 50	P. pachastrellae JCM 12285	304	387	High	Putative IIa	-
GCA_900105255.1	LT629763.1 218	P. sabulinigri JCM 14963	289	142.6	Low	Putative III	-
GCA_900105255.1	LT629763.1 219	P. sabulinigri JCM 14963	289	146.2	low	Putative III	-
GCA_900105255.1	LT629763.1 2628	P. sabulinigri JCM 14963	303	382	High	Putative IIa	-
GCA_900105255.1	LT629763.1 2629	P. sabulinigri JCM 14963	305	383.4	High	Putative IIa	-
GCA_900105655.1	LT629787.1 958	P. salegens CECT 8338	289	140.3	Low	Putative III	-

GCA_900105655.1	LT629787.1 957	P. salegens CECT 8338	288	140.7	Low	Putative III	-
GCA_900105655.1	LT629787.1 2802	P. salegens CECT 8338	315	383.5	High	Putative IIa	-
GCA_900105655.1	LT629787.1 2801	P. salegens CECT 8338	315	392.2	High	Putative IIa	-
GCA_014637955.1	BMFF01000001.1 657	P. salina CGMCC 1.12482	293	134.2	Low	Putative III	-
GCA_014637955.1	BMFF01000001.1 656	P. salina CGMCC 1.12482	291	134.6	Low	Putative III	-
GCA_014637955.1	BMFF01000001.1 582	P. salina CGMCC 1.12482	308	386.7	High	Putative IIa	-
GCA_900104945.1	LT629736.1 1619	P. xinjiangensis NRRL B-51270	285	142.6	Low	Putative III	-
GCA_900104945.1	LT629736.1 1618	P. xinjiangensis NRRL B-51270	287	151.6	Low	Putative III	-
GCA_900104945.1	LT629736.1 889	P. xinjiangensis NRRL B-51270	303	405.3	High	Putative IIa	-
GCA_900114825.1	FOUI01000001.1 410	P. yangmingensis DSM 24213	286	134.6	Low	Putative III	-
GCA_900114825.1	FOUI01000004.1 1	P. yangmingensis DSM 24213	303	380.7	High	Putative IIa	-
GCA_900104945.1	LT629736.1 1620	P.xinjiangensis NRRL B-51270	288	125.2	Low	Putative III	-

Table S5: Primer pairs for the introduction of single mutations in *Halo*PETase-1 by site-directed mutagenesis.

Primer	Sequence (5'→3')	Purpose
T88Y forward	caccctataatagtctggggtaatggatatacagcatcgccaagta	Restoring the π-stacking clamp
T88Y reverse	tacttggcgatgctgtatatccattaccccagactattatagggtg	
T88W forward	caccctataatagtctggggtaatggatggacagcatcgccaagta	
T88W reverse	tacttggcgatgctgtccatccattaccccagactattatagggtg	
T88F forward	caccctataatagtctggggtaatggatttacagcatcgccaagta	
T88F reverse	tacttggcgatgctgtccatccattaccccagactattatagggtg	
Q157M forward	gctggccatagcatgggcggcggcggc	Introduction of canonical methionine in lipase-box motif
Q157M reverse	gccgccgccgcccatgctatggccagc	

Supplementary references

1. Benson, D. A. *et al.* GenBank. *Nucleic Acids Research* **41**, D36–D42 (2013).
2. Altschul, S. F., Gish, W., Miller, W., Myers, E. W. & Lipman, D. J. Basic local alignment search tool. *Journal of Molecular Biology* **215**, 403–410 (1990).
3. Bollinger, A. *et al.* A Novel Polyester Hydrolase From the Marine Bacterium Pseudomonas aestusnigri – Structural and Functional Insights. *Front. Microbiol.* **11**, 114 (2020).
4. Avilan, L. *et al.* Concentration-Dependent Inhibition of Mesophilic PETases on Poly(ethylene terephthalate) Can Be Eliminated by Enzyme Engineering. *ChemSusChem* **16**, e202202277 (2023).
5. Larkin, M. A. *et al.* Clustal W and Clustal X version 2.0. *Bioinformatics* **23**, 2947–2948 (2007).
6. Gonnet, G. H., Cohen, M. A. & Benner, S. A. Exhaustive Matching of the Entire Protein Sequence Database. *Science* **256**, 1443–1445 (1992).
7. Joo, S. *et al.* Structural insight into molecular mechanism of poly(ethylene terephthalate) degradation. *Nature Communications* **9**, 382–382 (2018).
